# Patient Benefit-Risk Tradeoffs for Radioactive Iodine-Refractory Differentiated Thyroid Cancer Treatments

**DOI:** 10.1155/2015/438235

**Published:** 2015-11-30

**Authors:** Ateesha F. Mohamed, Juan Marcos González, Angelyn Fairchild

**Affiliations:** ^1^Bayer Healthcare Pharmaceuticals Inc., Whippany, NJ 07981, USA; ^2^RTI Health Solutions, Research Triangle Park, NC 27709, USA

## Abstract

*Background*. The aims of this study were to assess patients' preferences to wait or start systemic treatment and understand how patients would make tradeoffs between certain severe adverse events (AEs) and additional months of progression-free survival (PFS).* Materials and Methods*. Adults in France, Germany, and Spain with a diagnosis of DTC and who have had at least one RAI treatment completed a direct-elicitation question and a discrete-choice experiment (DCE) online. The direct-elicitation question asked respondents whether they would opt out of treatment when their tumor is RAI-R. In the DCE, respondents chose between 12 pairs of hypothetical RAI-R DTC treatment profiles. Profiles were defined by magnitudes of efficacy (PFS) and safety (severe hand-foot skin reaction [HFSR], severe proteinuria, and severe hypertension). A main-effects random-parameters logit model was estimated.* Results*. 134 patients completed the survey. Most patients (86.6%) opted for treatment rather than “wait and see” decision. Patients placed a greater weight on the risk of severe hypertension than the risk of proteinuria and HFSR.* Conclusions*. DTC patients showed preference toward treatment for RAI-R DTC over watchful waiting. Patients' concerns about the risk of severe hypertension appeared to have had a greater effect on patients' choice than severe proteinuria or HFSR.

## 1. Introduction

Worldwide, thyroid cancer accounts for 2.1% of all new cancers [1]. Differentiated thyroid cancer (DTC), which includes papillary, follicular, and Hürthle cell types, accounts for nearly 94% of thyroid cancers [2]. The main therapeutic approaches for DTC are surgical resection, radioactive iodine (RAI [^131^I]) ablation, and thyroid-stimulating hormone suppression [3, 4]. The overall prognosis for DTC is excellent with a 10-year disease-specific survival rate of 85% [5]. Approximately 10%–15% of patients develop distant metastases with a 10-year disease-specific survival rate of 40% [6]. However, for some DTC patients who develop metastases, the ability to uptake RAI is lost (i.e., the patients become RAI-refractory [RAI-R]) with a 10-year disease-specific survival rate of 10% [7].

Consensus is emerging on how to best define RAI-R DTC. It is defined in patients with advanced disease either by the presence of at least one tumor focus without any uptake of RAI, or by progression of the disease during the year after a course of treatment with RAI, or by persistent disease after the administration of a cumulative activity of 22 GBq (600 mCi) radioiodine (based on individual assessment) [8]. At progression, not all patients who develop RAI-R DTC experience disease-related symptoms, and physicians are faced with a decision on when to start treatment [9]. Conventional chemotherapeutic agents like doxorubicin have been used to treat RAI-R DTC with poor results and weak evidence support [3, 4, 10–12].

There has been research conducted on the identification of intracellular pathways involved in pathogenesis of DTC [13]. The focus is now on molecular targets like tyrosine kinase inhibitors (TKIs) and angiogenesis pathways [9, 14, 15]. Recently, sorafenib and lenvatinib were both approved for the treatment of RAI-R DTC based on positive randomized clinical trials [16, 17]. Currently, there is no head-to-head comparison study of these two approved treatments, which makes it difficult for physicians to decide between these two systemic treatments. There are currently no published studies evaluating patient preferences regarding treatment decisions for RAI-R DTC patients.

The aims of this study were to assess patients' preferences to wait or start systemic treatment and to understand how patients would make tradeoffs between additional months of progression-free survival (PFS) and certain severe adverse events (AEs) that differ between the two approved systemic treatments. The hypothesis is that when choosing treatments, patients consider long-term AEs with uncertain sequelae to be more important than short-term AEs that could lead to a worsening quality of life.

## 2. Materials and Methods

### 2.1. Preference-Elicitation Questions

We followed good practice [18] in designing and administering a discrete-choice experiment (DCE) to elicit patient preferences for RAI-R DTC treatments. This method is grounded in both psychology [19] and economics [20] and has been commonly applied in health [21, 22]. Several DCE studies in oncology have evaluated patient treatment preferences using online surveys [23–26]. DCE studies require respondents to answer a series of choice questions where they indicate which of several hypothetical treatment alternatives they prefer. Treatment alternatives are defined by the levels to which they satisfy various treatment attributes. The attribute levels are systematically varied across choice questions, generating treatment profiles that are not representative of any existing treatment [21–27]. Multinomial regression analysis of the respondents' choices results in the relative importance of a particular treatment as a function of the attribute levels included [22, 28].

A direct-elicitation question was included in the series of choice questions asking respondents to state whether they would opt out of treatment if their tumor was RAI-R. Respondents' preferences for treatment are represented by the proportion of respondents who would accept starting any of the treatments offered in the direct-elicitation question [19, 20].

### 2.2. Study Sample

Respondents who were at least 18 years old with a diagnosis of DTC and who had previously had at least one RAI treatment were recruited through medical clinics in France, Germany, and Spain (current use of systemic treatment was not an inclusion criterion). Respondents were invited to participate in the 25-minute online survey in February 2015. Each respondent was paid €30 in France and €25 in Germany and Spain as compensation for his or her time and inconvenience. The Office of Research Protection and Ethics at RTI International (Research Triangle Park, North Carolina, USA) approved this study, and respondents were required to provide online informed consent before participating in the survey.

### 2.3. Survey Instrument

To determine the four attributes and accompanying levels for the choice questions, we reviewed package inserts and phase 3 clinical trial data of recently approved systemic treatments [16, 17]. We included a main efficacy measure (months of PFS) and three main safety measures (grade 3/4 AEs): risk of severe hand-foot skin reaction (HFSR), risk of severe proteinuria, and risk of severe hypertension. The three severe AEs were chosen based on the severe AEs with the largest difference (at least 10%) in incidence rates reported in the phase 3 clinical trial data for the two approved TKIs [16, 17]. The levels for each attribute were designed to encompass the range observed in clinical trials and the range over which respondents were willing to make tradeoffs among the four attributes (Table 1). The definition for each attribute was presented using nontechnical language [18].

To assess the validity of the survey instrument, a draft version was tested in 15 face-to-face semistructured interviews in November, 2014, after which minor changes were made to the wording to improve respondent comprehension. During these interviews, patients were asked to “think aloud” as they completed the draft survey instrument and a series of debriefing questions to ascertain that they understood the attribute definitions, accepted the hypothetical context of the survey, and were able to complete the choice questions as instructed [18].

In each choice question, patients were asked to choose between two hypothetical treatment profiles (Table 2). Each profile was defined by the levels of the four attributes that varied in a systematic way (i.e., the experimental design). The experimental design was a main-effects D-efficient experimental design consisting of 36 choice questions and generated using SAS version 9.3 (SAS, Cary, North Carolina, USA) [28, 29]. The 36 choice questions were blocked into three sets of 12 choice questions, and respondents were randomly assigned to each block. Within each block, the order of the 12 choice questions was varied to control for potential order effects [28, 29]. In addition to the choice questions, the survey included demographic and disease-experience questions, a risk tutorial to assist patients in understanding the AE risk levels included, and a direct-elicitation question (within the series of choice questions) to determine if patients would opt to start systemic treatment and avoid the severe treatment-related AEs rather than to “wait and see” if their tumor progressed in the way expected from RAI-R DTC.

### 2.4. Statistical Analysis

Responses to the choice questions were analyzed using a random-parameters logit model [30–32]. The dependent variable was the treatment choice, and the explanatory variables were the attribute levels. All of the attributes listed in Table 1 were included in the model as continuous variables, where nonlinear effects were approximated with higher-order polynomial terms. Specification tests determined that preferences for improvements in PFS and severe hypertension changed nonlinearly and were modeled with quadratic and linear terms. Therefore, a one-unit change in each of these two attributes could have a different impact on preferences depending on the initial point of that improvement. The resulting parameter estimates quantified the relative strength of preference or preference weight of each attribute level [18, 24, 26, 27]. All analyses were conducted using NLOGIT 4.0 (Econometric Software, Inc., Plainview, New York, USA).

Results from the analysis of the choice questions were used to estimate patients' stated risk tolerance, or maximum acceptable risk (MAR), that would be tolerated for improvements in PFS. MAR is the mean maximum level of treatment-related risk patients are willing to accept for a given improvement in treatment benefit as inferred from responses to the choice questions. It is calculated as the change in the risk of a given severe AE (HFSR, proteinuria, or hypertension) that would exactly offset the perceived benefit of a given improvement in PFS [26, 27].

## 3. Results

### 3.1. Patients Sample Characteristics

Of the 162 patients invited to participate, 144 responded to the invite and 141 were eligible. Of the eligible respondents, 134 (response rate = 82.7%) provided informed consent and were included in the final analysis, which is a sample size consistent with current DCE practices in health [21].  Table 3 summarizes the demographic characteristics of the final sample: 84% were female, 78% were married, 58% were employed, 87% had papillary thyroid cancer, and 68% were diagnosed at least 2 years ago; the mean (standard deviation [SD]) age was 47.2 (12.5) years. Nearly 20% of the sample (19.4%) reported having high blood pressure, though no information was available on the severity of this health problem or whether it was attributable to DTC medications. Of the patients who completed the survey, 8.2% stated they were on systemic therapy.

### 3.2. Patient Preferences

**Figure 1:**
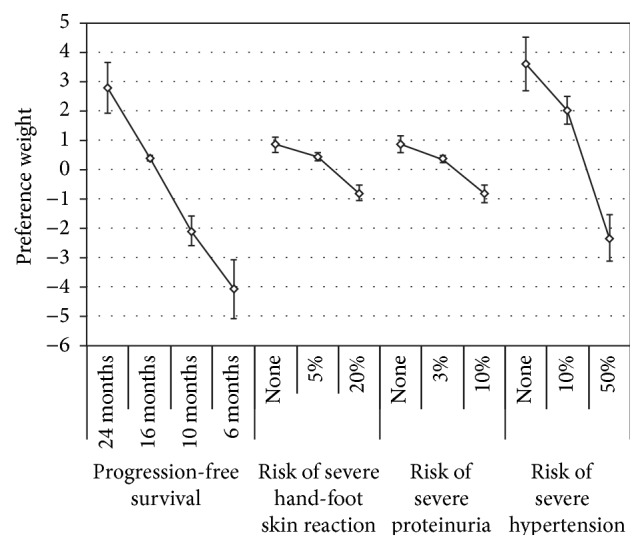
Preference weights (*N* = 134). Only relative differences matter when interpreting preference weights. The differences between adjacent preference weights indicate the relative impact of moving from one level of an attribute to an adjacent level of that attribute. Note: the vertical lines around each mean preference weight denote the 95% confidence interval about the point estimate.

Most patients (86.6%) opted for treatment rather than “waiting and seeing” if their tumor progressed in the way expected from RAI-R DTC.  Figure 1 presents the estimated preference weights and 95% confidence intervals (CIs) for the four attributes. The mean estimates were ordered as expected (i.e., better clinical outcomes had higher estimates) and were statistically significantly different (*p* < 0.05) between all adjacent levels for all four attributes.

Only relative differences matter when interpreting preference weights. The differences between adjacent preference weights indicate the relative impact of moving from one level of an attribute to an adjacent level of that attribute; the greater the difference, the more significant the change from one level to the next. For example, the relative impact of moving from 6 months of PFS to 10 months of PFS was approximately 1.97 (−2.11 − [−4.08]).

Similarly, the relative impact of a specific change in one attribute can be compared with the relative impact of a specific change in another attribute to understand whether the magnitude of the impact of a given change was comparable across attributes. For example, the relative impact of moving from 0% to 10% on severe proteinuria (1.68) was approximately 2 times the relative impact of moving from 0% to 10% on severe HFSR (0.83). As both of these variables were linear, the implication is that a 1%-point increase in the risk of severe proteinuria was twice as impactful to patients as a 1%-point increase in the risk of severe HFSR.

The vertical distance between the preference weights for the best and worst levels of any attribute indicates the overall relative importance of that attribute. Over the range of attributes and levels included in the survey, respondents considered improving PFS from 6 months to 24 months (i.e., improving PFS by 18 months) to be the most important attribute. Reducing the treatment-related risk of severe hypertension from 50% to none was approximately 0.86 times as important as improving PFS by 18 months. Improving the treatment-related risk of severe HFSR from 20% to none was approximately equally as important as improving the treatment-related risk of severe proteinuria from 10% to none; these changes were approximately 0.24 times and 0.25 times as important as improving PFS by 18 months, respectively. Among the three severe AEs shown, and given the ranges of risk presented to patients, greater weight was assigned to hypertension than the risk of proteinuria and HFSR.

### 3.3. Stated Risk Tolerance

Table 4 lists the MARs associated with improving PFS from 10 months to 16 months and improving PFS from 10 months to 18 months, respectively. For example, for an 8-month improvement in PFS, the maximum tolerated risk (i.e., prevalence) for severe hypertension was 21.8% (95% CI: 16.0%–27.7%), for severe proteinuria was 18.8% (95% CI: 12.9%–24.8%), and for severe HFSR was 38.5% (95% CI: 27.6%–49.3%). The 8-month improvement was clinically relevant, as the difference in the median PFS reported in the phase 3 clinical trial data for the two approved TKIs was approximately 7.5 months [16, 17].

## 4. Discussion

Our study had three main findings and potential clinical implications. First, DTC patients showed preference toward treatment for RAI-R DTC over watchful waiting given the tradeoffs offered in the direct-elicitation question. Under this scenario, 86.6% of patients opted to start treatment rather than to “wait and see,” as patients understood that once DTC progresses to RAI-R, it is no longer a slow-moving disease [5–7]. On the other hand, being RAI-R DTC usually means that the patients have undergone a number of previous and ultimately unsuccessful treatments, which may impact the decision to start a new treatment when they can observe the outcome of their disease in response to treatment.

Second, our study indicated that patients had clear preferences among the four selected treatment-related benefits and risks of RAI-R DTC treatments and traded off among them when choosing a treatment. This adds to the existing literature in RAI-R DTC, as there are currently no available data on patients' treatment preferences. Patients' perspectives can be considered in shared decision making between patients and physicians. Studies like this one also can offer some patient insights into aspects of treatment versus “wait and see” decision.

Third, patients valued improvement in PFS as the most important attribute. However, patients' concerns about the risk changes included in this study for severe hypertension appeared to have had a greater impact on patients' choice of treatment than the changes included for the risks of severe proteinuria or severe HFSR. Potential explanations for this finding came from the face-to-face interviews where patients mentioned that they were more concerned about AEs that had no short-term symptoms but that could result in potentially serious sequelae like life-threatening cardiac events due to chronic hypertension or renal impairment due to proteinuria. It is possible that patients were concerned that these AEs may require regular monitoring and may cause permanent health problems. Although bothersome and painful, onset of HFSR is evident to patients and the symptoms may be transient, which may give patients more control of the event. This information from the patients could help us understand patients' perspectives on these three common AEs and suggest areas for discussion between patients and physicians to make a treatment decision for RAI-R DTC.

Although DCE studies are increasingly used in health applications, they have limitations. First, respondents evaluate hypothetical treatments; although the tradeoffs are intended to simulate possible clinical decisions, they do not have the same clinical, financial, or emotional consequences of actual decisions. Thus, differences can arise between stated and actual treatment choices. Second, this study included only the selective AEs that differed between the two approved systemic therapies. There may be other factors that can influence actual treatment decisions that are not accounted for in this study.

Third, our sampling strategy within the study design limits the confidence with which these results can be generalized to the RAI-R DTC patient population. For example, we surveyed a convenience sample of DTC patients in France, Germany, and Spain with access to the Internet. Our sample was younger and had more females compared with the actual patient populations in the clinical trials [16, 17]. Our sample included a small proportion (8.2%) of RAI-R DTC patients on systemic therapy; therefore, a portion of patients who participated in this study did not have experience with RAI-R disease and would not have been exposed to treatment-related risks of the three AEs included in the study. Although our study was not powered to test for variations in preferences between subgroups of respondents, it is unclear whether these differences mattered as previous preference studies have found that patient characteristics or experiences do not always have an effect on treatment preferences [33, 34]. Nevertheless, caution should be exercised when trying to generalize our findings to patients with different demographic or treatment histories or to patients in other countries in Europe, or elsewhere. For example, the finding that PFS was the most important attribute and severe hypertension was more important than severe proteinuria or severe HFSR may have a different impact on an older sample of actual RAI-R DTC patients who may have other comorbidities and can better understand the impact of comorbidities such as severe hypertension in their lives. Future research using a randomized patient sample being treated with TKIs for RAI-R DTC to verify our findings would be particularly valuable.

In conclusion, DTC patients showed preference toward treatment for RAI-R DTC over watchful waiting. Patients' concerns about the risk of severe hypertension appeared to have had a greater impact on patients' choice of systemic treatment than concerns about severe proteinuria or severe HFSR. The results of this study may offer some insights into patients' perspectives on treatments and offer some guidance in shared decision making between patients and physicians for RAI-R DTC treatments.

## Figures and Tables

**Table 1:** Attributes and levels included in the final survey instrument.

Attribute	Attribute definition	Levels
Time until cancer grows (progression-free survival)	One of the most important goals of cancer medicines is to keep the tumor from getting worse. Later in the survey, we will ask you to think about how long different medicines can keep the tumor from growing or getting worse.	24 months 16 months 10 months 6 months

Risk of severe hand-foot skin reaction because of the medicine	Some medicines to treat thyroid cancer may cause severe hand-foot skin reactions. Severe hand-foot skin reactions cause redness, pain, swelling, or blisters on the palms of your hands or soles of your feet. This type of skin reaction makes it difficult to walk or use your hands. If you get this side effect, your doctor may change your dose or stop treatment for a period of time.	None 5 out of 100 (5%) 20 out of 100 (20%)

Risk of severe kidney problems (proteinuria) because of the medicine	Some medicines to treat thyroid cancer may *cause problems with your kidneys*. The kidneys are organs that filter your blood and remove waste from it. This waste is released as urine. When people have kidney problems, proteins can leak from the blood into the urine. This is known as *proteinuria*. Other symptoms of kidney problems include swelling of your arms and legs, poor appetite, and weight gain. If you have severe kidney problems, your doctor may change your dose or stop treatment for a period of time to reduce the risk that you will have permanent kidney damage.	None 3 out of 100 (3%) 10 out of 100 (10%)

Risk of severe high blood pressure (hypertension) because of the medicine	Some medicines to treat thyroid cancer may cause *severe high blood pressure*. When people have severe high blood pressure, they may experience severe headaches, tiredness that cannot be relieved by sleeping, vision problems, chest pain, and difficulty in breathing. If you have severe high blood pressure, your doctor may change your dose or stop treatment for a period of time to reduce the risk that you have a heart attack or a stroke.	None 10 out of 100 (10%) 50 out of 100 (50%)

**Table 2:** Example choice question.

Medicine feature	Medicine A	Medicine B
Time until tumor grows	10 months	24 months
Risk of severe hand-foot skin reaction because of medicine	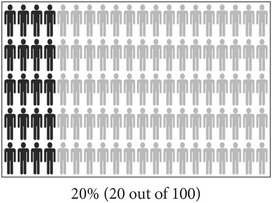	None
Risk of severe kidney problems because of medicine	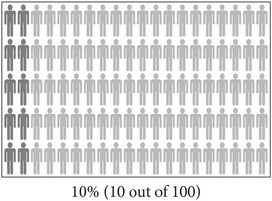	None
Risk of severe high blood pressure because of medicine	None	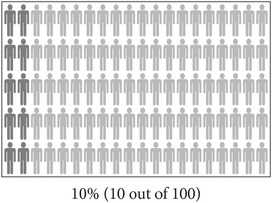
If these were the only alternatives available, which medicine would you choose?	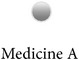	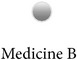

Each respondent answered 12 choice questions. This is just one example from the full set of 36 choice questions.

**Table 3:** Summary of patient characteristics.

Question	Number (%) *N* = 134
What is your gender?	
Male	21 (15.7%)
Female	113 (84.3%)
What is your age? Mean (SD) years	47.2 (12.5)
What is your marital status?	
Single/never married	16 (11.9%)
Married/living as married/civil partnership	104 (77.6%)
Divorced or separated	9 (6.7%)
Widowed/surviving partner	5 (3.7%)
Which of the following best describes your employment status?	
Employed full-time/part-time/self-employed	78 (58.2%)
Homemaker/student/retired	40 (29.9%)
Disabled/unable to work/unemployed	16 (11.9%)
What type of health insurance do you have?	
Public health insurance only	84 (62.7%)
Private health insurance	49 (36.6%)
Other	1 (0.7%)
Which of the following have you been told by a doctor or another health care provider that you have or have had?^a^	
Papillary thyroid cancer	117 (87.3%)
Follicular thyroid cancer	15 (11.2%)
Follicular variant of papillary thyroid cancer	1 (0.7%)
Medullary thyroid cancer	3 (2.2%)
Which of the following treatments have you used for your thyroid cancer?^a^	
Surgery	121 (90.3%)
Radioactive iodine	134 (100.0%)
Thyroid-stimulation hormone (TSH) suppression	49 (36.6%)
External beam radiation therapy (EBRT)	2 (1.5%)
Pills or tablets to stabilize or reduce the tumor size (systemic therapy)	11 (8.2%)
Other	4 (3.0%)
Approximately how long ago were you originally diagnosed with thyroid cancer?	
Less than 2 years ago	43 (32.1%)
At least 2 years ago but less than 5 years ago	41 (30.6%)
At least 5 years ago	50 (37.3%)
Are you currently being treated for thyroid cancer tumors (excluding screening or regular monitoring)?	
Yes	32 (23.9%)
No	102 (76.1%)
How many times has your doctor had you complete a radioactive iodine treatment to treat your cancer?	
1	83 (61.9%)
2	40 (29.9%)
3	7 (5.2%)
More than 3	4 (3.0%)
Have you ever been diagnosed with high blood pressure?	
Yes	26 (19.4%)^b^
No	108 (80.6%)

SD = standard deviation.

^a^Respondents can tick more than one answer.

^b^16 (61.5%) patients with high blood pressure were taking medicine to treat their high blood pressure.

**Table 4:** Maximum acceptable risks.

Grade 3/4 adverse event	6-month improvement in PFS from 10 months to 16 months (95% CI)	8-month improvement in PFS from 10 months to 18 months (95% CI)
Severe hand-foot skin reaction	30.0% (21.5%–38.5%)	38.5% (27.6%–49.3%)
Severe proteinuria	14.7% (10.0%–19.4%)	18.8% (12.9%–24.8%)
Severe hypertension	16.5% (11.9%–21.0%)	21.8% (16.0%–27.7%)

CI = confidence interval; PFS = progression-free survival.
